# Electron Microscopy Reveals Evidence of Perinuclear Clustering of Mitochondria in Cardiac Biopsy-Proven Allograft Rejection

**DOI:** 10.3390/jpm12020296

**Published:** 2022-02-17

**Authors:** Estefanía Tarazón, Lorena Pérez-Carrillo, Manuel Portolés, Esther Roselló-Lletí

**Affiliations:** 1 Myocardial Dysfunction and Cardiac Transplantation Unit, Health Research Institute Hospital La Fe (IIS La Fe), 46026 Valencia, Spain; lorena_perezc@iislafe.es (L.P.-C.); portoles_man@gva.es (M.P.); 2 CIBERCV, 28029 Madrid, Spain

**Keywords:** mitochondria, endomyocardial biopsy, cardiac rejection, electron microscopy

## Abstract

Acute cellular rejection is a major complication in heart transplantation. We focus on the analysis of new ultrastructural findings in cardiac biopsy rejection based on mitochondrial intracellular organization. This study includes heart transplanted patients from a single center who were referred for endomyocardial biopsies as a scheduled routine screening. Participants were divided into two groups: patients transplanted without allograft rejection (Grade 0R), and patients with biopsy-proven allograft rejection (Grade ≥ 2R). Using electronic microscopy, we detected a significant increase in the volume density of mitochondria (*p* < 0.0001) and dense bodies (*p* < 0.01) in the rejection group. The most relevant finding was the presence of local accumulations of mitochondria close to the nuclear envelope, pressing and molding the morphology of this membrane in all rejection samples (100%). We identified this perinuclear clustering of mitochondria phenomenon in a 68 ± 27% of the total cardiac nucleus observed from rejection samples. We did not observe this phenomenon in any non-rejection samples, reflecting excellent sensitivity and specificity. We have identified a specific phenomenon affecting the architecture of the nuclear membrane—perinuclear clustering of mitochondria—in endomyocardial biopsies from patients with cardiac rejection. This ultrastructural approach might complement and improve the diagnosis of rejection.

## 1. Introduction

Heart transplantation is a therapy that contribute to an improved quality of life and longevity of patients with advanced heart failure. Despite improved efficacy and selection of immunosuppression therapy over the last few years, allograft rejection continues to be a significant risk, especially early after transplantation [1].

Endomyocardial biopsy (EMB) is the standard clinical tool with a recognized role in the surveillance of post-transplant cardiac rejection and is based on optical microscopy analysis [2]. This method presents important technical limitations, such as the low sensitivity of the standardized determinations that are currently performed. Thus, the conventional histological interpretation of EMB is inaccurate. Non-invasive methods and reliable biomarkers are actively sought to screen for heart transplant rejection [3,4,5,6,7,8,9], but unfortunately EMB is nowadays the gold standard. Therefore, it is necessary improve this histological analysis. Electron microscopy allows complete observation of cardiac cellular damage and subcellular changes in heart diseases as have been shown in numerous studies [10,11,12]. However, the significance of ultrastructural alterations in cardiomyocytes from patients with cardiac rejection has not been investigated thoroughly.

The importance of mitochondria in cardiovascular pathophysiology has been increasingly recognized over the past decades and its role is receiving ever-growing attention [13,14,15]. We previously showed that mitochondrial alterations are reflected in peripheral blood and are capable of discriminating between patients with allograft rejection and those not experiencing rejection with excellent accuracy [6].

In this work we focus on the analysis of new ultrastructural findings on cardiac biopsy specimens that provides additional information to improve the diagnosis of cardiac rejection. Considering the invasiveness of the standard procedure, it is critical to make better use of the cardiac sample taken. This ultrastructural approach based on mitochondrial intracellular organization changes may improve the limited diagnosis of cardiac rejection.

## 2. Materials and Methods

A total of 10 EMBs were collected from heart transplant patients (>18-years) who were referred for EMB for a scheduled routine screening at the University and Polytechnic Hospital La Fe. Of the patients studied, 5 had a diagnosis of biopsy-proven allograft rejection (grade ≥ 2R). These samples were compared with samples from the 5 patients who did not experience allograft rejection. The associated clinical data were also collected from follow-up visits of the cardiac transplant recipients.

This study was approved by the Ethics Committee (Biomedical Investigation Ethics Committee of La Fe University Hospital of Valencia, Valencia, Spain) and was conducted in accordance with the guidelines of the Declaration of Helsinki [16]. Prior to sample collection, signed informed consent was obtained from each patient.

Tissue processing for electron microscopy was performed as described previously [11]. Briefly, left ventricular (LV) samples (size 0.5 mm^3^) were fixed for 1 h at 4 °C in a solution of 1.5% glutaraldehyde and 1% formaldehyde in a 0.05 M cacodylate buffer (pH 7.4). Later, the samples were post-fixed in buffered 1% osmium tetroxide (OsO_4_) for 1 h at 4 °C, dehydrated in a series of ethanol solutions, and embedded in Epon 812. Semi-thin sections were first evaluated with a light microscope (Olympus BX-50, Olympus, Tokyo, Japan) before moving onto the evaluation of ultra-thin sections (Ultramicrotome Leica EM UC6, Leica Microsystems, Wetzlar, Germany). These sections (80 nm) were obtained and mounted on copper grids and subsequently counter-stained with 2% uranyl acetate for 20 min and 2.7% lead citrate for 3 min. The samples were observed using a JEOL JEM-1010 electron microscope and analyzed in a specific program (iTEM FEI program, v.5.0, 2008, Olympus Soft Imaging Solutions GmbH, Münster, Germany). The selection of the image study areas was carried out with the systematic random sampling method [17,18,19]. The mitochondria and dense bodies’ volume densities were measured according to the stereological methods previously described [20]. For the counting of mitochondria and dense bodies of samples analyzed in the perinuclear area (the same area for each micrograph, 29.97 µm^2^), a randomized point grid was superimposed on the images obtained by transmission electron microscopy. After counting points in an image, we obtained the number of points that fall in the mitochondria or dense body and the number of points in the reference space. The data were expressed as the percentage of cytoplasm occupied by mitochondria or dense bodies [21]. The ImageJ grid tool was used for point randomization.

Results for each variable were tested for normality using the Kolmogorov–Smirnov method. Continuous variables are presented as mean ± standard deviation (variables with normal distribution) or median and interquartile range (non-normal distributions), and categorical variables as percentage. Continuous variables not following a normal distribution were compared using the Mann–Whitney test and variables with a normal distribution were compared using the Student’s t-test. Fisher’s exact test was used to compare discrete variables. All statistical analyses were performed using SPSS software v. 20, 2012 for Windows (IBM SPSS Inc., Chicago, IL, USA). Significance was accepted at the *p* < 0.05 level.

## 3. Results

This study includes heart transplanted patients (>18 years) from a single center who were referred for EMB as a scheduled routine screening. Study participants were divided into two groups: patients transplanted without acute cellular rejection (Grade 0R, *n* = 5), and patients with biopsy-proven acute cellular rejection (Grade ≥ 2R, *n* = 5). Patients were maintained on a standard immunosuppression regime, and rejection episodes were assessed according to the International Society for Heart and Lung Transplantation (ISHLT) consensus report [22].

For each sample, we recorded age, gender, body mass index, primary heart disease, interval between transplantation and study enrollment, biochemical markers, echocardiographic parameters, and other clinical characteristics at the time of each biopsy (Table 1). Both populations of the study had similar clinical characteristics. The rejection and non-rejection groups were similar with regard to variables such as age, sex, body mass index, hypertension, immunosuppressive and induction therapy, hemoglobin concentration and hematocrit percentage, among others. However, we found an increase in lymphocyte number in the rejection group and also an increase in the N-terminal pro–B-type natriuretic peptide (NT-proBNP) levels, but it did not reach statistical significance.

**Figure 1:**
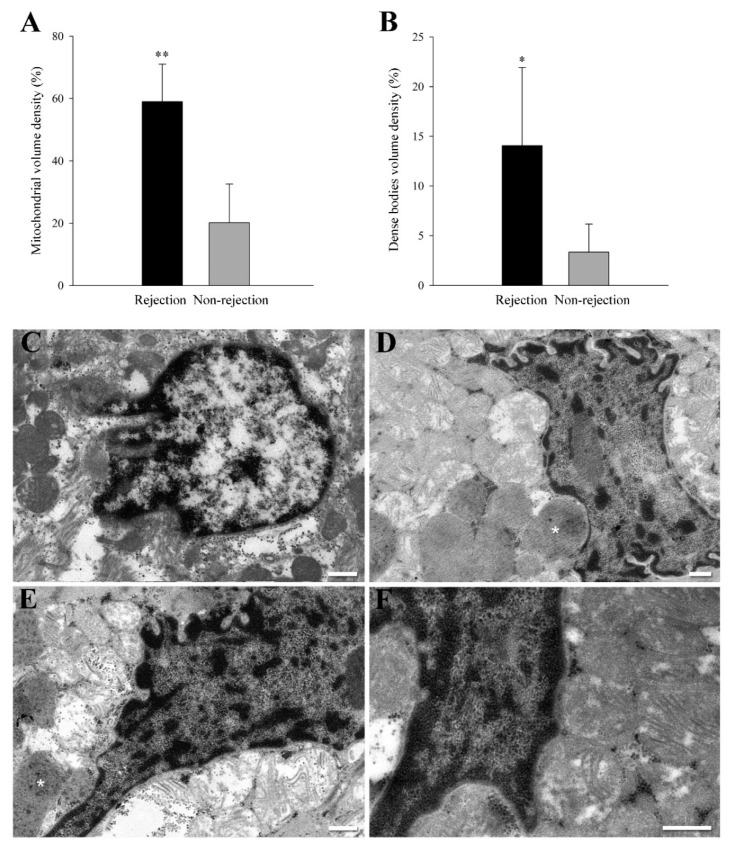
Ultrastructural evidence of perinuclear clustering of mitochondria in acute cellular rejection. (**A**,**B**) mitochondria and dense bodies volume density in a total of 300 randomly selected micrographic areas (30 areas per sample). Bars indicate the % of cytoplasm occupied by mitochondria or dense bodies^2^ ± SD (standard deviation) in the perinuclear area. * *p* < 0.01 and ** *p* < 0.0001 against the non-rejection group. (**C**) Representative transmission electron microscopy micrograph from a non-rejection cardiac tissue sample. (**D**–**F**) Representative transmission electron microscopy micrograph from rejection cardiac tissue samples. These images show a specific phenomenon found in rejection samples and not observed in the non-rejection samples. These sections show the presence of local accumulations of mitochondria close to the nuclear envelope pressing and molding the morphology of this membrane. White asterisks indicate other structures of interest: dense bodies corresponding to mitochondria in the degradation process. White bars represent 400 nm.

We analyzed the volume density of mitochondria and degradation structures (dense bodies) in a total of 300 randomly selected micrographic areas (30 areas per sample). After conducting an exhaustive ultrastructural analysis [20], we detected a significative increase in the mitochondrial volume density (59 ± 12% vs. 20 ± 12%, *p* < 0.0001) (Figure 1A) and dense bodies volume density (14 ± 8% vs. 3 ± 3%, *p* < 0.01) (Figure 1B) in the rejection group. The dense bodies quantified are mitochondria in degradation process. Nevertheless, the most significant finding was the changes in mitochondrial location and distribution on sections obtained from biopsies of rejection patients. We did not find this phenomenon in any of the non-rejection samples analyzed (Figure 1C), reflecting an excellent sensitivity and specificity of the effect detected. In all the samples from rejection patients (100%), we identified the presence of local accumulations of mitochondria close to the nuclear envelope, pressing and molding the morphology of this membrane (Figure 1D–F). We found perinuclear clustering of mitochondria in a 68 ± 27% of the total cardiac nuclei observed in rejection samples.

## 4. Discussion

Preventing and treating rejection is a crucial challenge in heart transplantation because it is a major complication [23,24]. Therefore, deeper knowledge of the mechanisms preceding rejection and an appropriate diagnosis in early stages is required to further improve the survival of transplanted patients. EMB is the reference process for detection of allograft rejection and the stained tissue samples are graded using the ISHLT classification [22]. The main criterion for acute cellular rejection severity is the grade of mononuclear cell infiltration. However, this method is subject to interobserver variability, and this limitation could be moderate improving the microscopic examination.

Intranuclear mitochondria have occasionally been reported in several cell types, including cardiac myocytes [25,26], but the incidence and details regarding the specific mechanism whereby mitochondria appear in the nuclear matrix are unknown. In this study, we did not find any mitochondria inside the nucleus; only we found this organelle in the perinuclear area. However, it remains possible that we are observing the first step of the mitochondrial movement towards the nucleus. In fact, there are strong similarities between the phenomena we have observed and reported images showing the intranuclear location of mitochondria in the cardiomyocytes of patients with heart failure; these authors also found the same effect [25]. Previous reports have suggested that the mechanism underlying the intranuclear location of mitochondria and its distribution in clusters or networks could be due to the deposit of apoptosis-activating proteins that initiate the reaction cascade finally resulting in the degradation of nuclear DNA and nuclear proteins [27,28]. Considering the great diagnostic value of electron microscopic examination, it may be recommended that the fixation of a small piece of tissue for electron microscopy occur at every EMB procedure for detection of cardiac rejection.

The dense bodies appear as aggregates of matrix precipitates that are spherical or occasionally irregular in shape and sometimes indistinguishable from woolly densities [29]. Although the mechanisms that induce the appearance of dense bodies are unknown, among the hypotheses proposed is that the increase in oxidative stress can cause defects in mitochondrial metabolism, promoting the appearance of dysfunctional mitochondria that contain non-degradable dense bodies [30,31]. In the context of several cardiac pathologies, accumulation of vacuoles of degeneration in the perinuclear space has been described, whose content was formed mainly by mitochondria, dense bodies, and other cellular components [32].

We show a preliminary study that may be performed in a larger patient cohort in order to better investigate this finding and lead to the standardization of this complementary determination in the detection of rejection. Taking into account the invasiveness of the methodology, it is essential to make better use of the cardiac sample obtained. This ultrastructural approach may improve the limited diagnosis of cardiac rejection.

Our study has several limitations, and the results must be interpreted within this context. This investigation only involved a single center, and the findings obtained should be assessed in a larger multicenter study. In addition, our study is focused on cellular rejection and has not specifically evaluated antibody-mediated rejection. However, we believe that our findings have provided substantial evidence and represent a necessary first step to support future research in which these limiting factors could be overcome.

## 5. Conclusions

In summary, we have observed by electron microscopy a specific phenomenon whereby mitochondria appear to cluster in a perinuclear fashion affecting the architecture of the nuclear membrane. We only identified this phenomenon in patients with cardiac allograft rejection. This ultrastructural approach could complement and improve the diagnosis of rejection, at least in the clinically relevant grades.

## Figures and Tables

**Table 1:** Patient characteristics at the time of biopsy.

	Non-Rejection (*n* = 5)	Rejection (*n* = 5)	*p*-Value
Age (years)	44 ± 18	41 ± 7	0.755
Male sex (%)	100	80	0.556
Indication for cardiac transplantation			
Ischemic cardiomyopathy (%)	50	80	0.722
Idiopathic dilated cardiomyopathy (%)	25	20	0.405
Other (%)	25	0	0.444
Time between transplantation and study enrolment, months	2.98 ± 2.94	2.55 ± 2.82	0.833
Hypertension (%)	25	40	0.595
Hemodynamic parameters			
Mean right atrial pressure (mmHg)	5.50 ± 3.32	5.67 ± 2.52	0.945
Systolic right ventricular pressure (mmHg)	36 ± 7	34 ± 4	0.647
Diastolic right ventricular pressure (mmHg)	6.00 ± 4.16	5.33 ± 2.08	0.812
Immunosuppressive therapy			
Tacrolimus (%)	100	100	-
Mycophenolic acid (%)	100	100	-
Steroids (%)	100	100	-
Induction therapy Basiliximab (%)	100	100	-
Neutrophils (thousands/mm^3^)	7.26 ± 3.87	11.42 ± 4.78	0.225
Leukocytes (thousands/mm^3^)	5.33 ± 3.25	7.53 ± 4.35	0.448
Lymphocytes (thousands/mm^3^)	1.14 ± 0.44	2.85 ± 1.24	0.041
Hemoglobin (mg/dL)	12.10 ± 3.83	11.33 ± 2.98	0.760
Hematocrit (%)	37 ± 11	36 ± 8	0.831
NT-proBNP (pg/mL)	111 ± 47	1441 ± 3449	0.083

NT-proBNP—N-terminal fragment of B-type natriuretic peptide.

## References

[1] Stehlik J., Edwards L.B., Kucheryavaya A.Y., Benden C., Christie J.D., Dipchand A.I., Dobbels F., Kirk R., Rahmel A.O., Hertz M.I. (2012). The Registry of the International Society for Heart and Lung Transplantation: 29th official adult heart transplant report—2012. J. Heart Lung Transplant. Off. Publ. Int. Soc. Heart Transplant..

[2] Nielsen H., Sorensen F.B., Nielsen B., Bagger J.P., Thayssen P., Baandrup U. (1993). Reproducibility of the acute rejection diagnosis in human cardiac allografts. The Stanford Classification and the International Grading System. J. Heart Lung Transplant. Off. Publ. Int. Soc. Heart Transplant..

[3] Crespo-Leiro M.G., Stypmann J., Schulz U., Zuckermann A., Mohacsi P., Bara C., Ross H., Parameshwar J., Zakliczynski M., Fiocchi R. (2016). Clinical usefulness of gene-expression profile to rule out acute rejection after heart transplantation: CARGO II. Eur. Heart J..

[4] Agbor-Enoh S., Shah P., Tunc I., Hsu S., Russell S., Feller E., Shah K., Rodrigo M.E., Najjar S.S., Kong H. (2021). Cell-Free DNA to Detect Heart Allograft Acute Rejection. Circulation.

[5] Perez-Carrillo L., Sanchez-Lazaro I., Trivino J.C., Feijoo-Bandin S., Lago F., Gonzalez-Juanatey J.R., Martinez-Dolz L., Portoles M., Tarazon E., Rosello-Lleti E. (2021). Diagnostic value of serum miR-144-3p for the detection of acute cellular rejection in heart transplant patients. J. Heart Lung Transplant. Off. Publ. Int. Soc. Heart Transplant..

[6] Tarazon E., Perez-Carrillo L., Garcia-Bolufer P., Trivino J.C., Feijoo-Bandin S., Lago F., Gonzalez-Juanatey J.R., Martinez-Dolz L., Portoles M., Rosello-Lleti E. (2021). Circulating mitochondrial genes detect acute cardiac allograft rejection: Role of the mitochondrial calcium uniporter complex. Am. J. Transplant. Off. J. Am. Soc. Transplant. Am. Soc. Transpl. Surg..

[7] Tarazon E., Corbacho-Alonso N., Barderas M.G., Gil-Cayuela C., Garcia-Manzanares M., Feijoo-Bandin S., Lago F., Gonzalez-Juanatey J.R., Martinez-Dolz L., Portoles M. (2020). Plasma CD5L and non-invasive diagnosis of acute heart rejection. J. Heart Lung Transplant. Off. Publ. Int. Soc. Heart Transplant..

[8] Tarazon E., Gil-Cayuela C., Manzanares M.G., Roca M., Lago F., Gonzalez-Juanatey J.R., Sanchez-Lacuesta E., Martinez-Dolz L., Portoles M., Rosello-Lleti E. (2019). Circulating Sphingosine-1-Phosphate as A Non-Invasive Biomarker of Heart Transplant Rejection. Sci. Rep..

[9] Tarazon E., Ortega A., Gil-Cayuela C., Sanchez-Lacuesta E., Marin P., Lago F., Gonzalez-Juanatey J.R., Martinez-Dolz L., Portoles M., Rivera M. (2017). SERCA2a: A potential non-invasive biomarker of cardiac allograft rejection. J. Heart Lung Transplant. Off. Publ. Int. Soc. Heart Transplant..

[10] Tarazon E., Rosello-Lleti E., Ortega A., Gil-Cayuela C., Gonzalez-Juanatey J.R., Lago F., Martinez-Dolz L., Portoles M., Rivera M. (2017). Changes in human Golgi apparatus reflect new left ventricular dimensions and function in dilated cardiomyopathy patients. Eur. J. Heart Fail..

[11] Cortes R., Rosello-Lleti E., Rivera M., Martinez-Dolz L., Salvador A., Azorin I., Portoles M. (2010). Influence of heart failure on nucleocytoplasmic transport in human cardiomyocytes. Cardiovasc. Res..

[12] Basso C., Czarnowska E., Della Barbera M., Bauce B., Beffagna G., Wlodarska E.K., Pilichou K., Ramondo A., Lorenzon A., Wozniek O. (2006). Ultrastructural evidence of intercalated disc remodelling in arrhythmogenic right ventricular cardiomyopathy: An electron microscopy investigation on endomyocardial biopsies. Eur. Heart J..

[13] Rosca M.G., Hoppel C.L. (2010). Mitochondria in heart failure. Cardiovasc. Res..

[14] Rosello-Lleti E., Tarazon E., Barderas M.G., Ortega A., Molina-Navarro M.M., Martinez A., Lago F., Martinez-Dolz L., Gonzalez-Juanatey J.R., Salvador A. (2015). ATP synthase subunit alpha and LV mass in ischaemic human hearts. J. Cell. Mol. Med..

[15] Kuznetsov A.V., Javadov S., Margreiter R., Grimm M., Hagenbuchner J., Ausserlechner M.J. (2019). The Role of Mitochondria in the Mechanisms of Cardiac Ischemia-Reperfusion Injury. Antioxidants.

[16] Macrae D.J. (2007). The Council for International Organizations and Medical Sciences (CIOMS) guidelines on ethics of clinical trials. Proc. Am. Thorac. Soc..

[17] Mouton P. (2002). Principles and Practices of Unbiased Stereology: An Introduction for Bioscientists.

[18] Baddeley A., Jensen E.B.V. (2005). Stereology for Statisticians.

[19] Howard V., Reed M. (2005). Unbiased Stereology.

[20] Duranova H., Valkova V., Knazicka Z., Olexikova L., Vasicek J. (2020). Mitochondria: A worthwhile object for ultrastructural qualitative characterization and quantification of cells at physiological and pathophysiological states using conventional transmission electron microscopy. Acta Histochem..

[21] Bozzola J.J., Russell L.D. (1999). Electron Microscopy.

[22] Stewart S., Winters G.L., Fishbein M.C., Tazelaar H.D., Kobashigawa J., Abrams J., Andersen C.B., Angelini A., Berry G.J., Burke M.M. (2005). Revision of the 1990 working formulation for the standardization of nomenclature in the diagnosis of heart rejection. J. Heart Lung Transplant. Off. Publ. Int. Soc. Heart Transplant..

[23] Lund L.H., Edwards L.B., Kucheryavaya A.Y., Dipchand A.I., Benden C., Christie J.D., Dobbels F., Kirk R., Rahmel A.O., Yusen R.D. (2013). The Registry of the International Society for Heart and Lung Transplantation: Thirtieth Official Adult Heart Transplant Report—2013; focus theme: Age. J. Heart Lung Transplant. Off. Publ. Int. Soc. Heart Transplant..

[24] Stoica S.C., Cafferty F., Pauriah M., Taylor C.J., Sharples L.D., Wallwork J., Large S.R., Parameshwar J. (2006). The cumulative effect of acute rejection on development of cardiac allograft vasculopathy. J. Heart Lung Transplant. Off. Publ. Int. Soc. Heart Transplant..

[25] Bakeeva L.E., Skulachev V.P., Sudarikova Y.V., Tsyplenkova V.G. (2001). Mitochondria enter the nucleus (one further problem in chronic alcoholism). Biochem. Biokhimiia.

[26] Eldarov C.M., Vangely I.M., Vays V.B., Sheval E.V., Holtze S., Hildebrandt T.B., Kolosova N.G., Popkov V.A., Plotnikov E.Y., Zorov D.B. (2020). Mitochondria in the Nuclei of Rat Myocardial Cells. Cells.

[27] Skulachev V.P. (2001). Mitochondrial filaments and clusters as intracellular power-transmitting cables. Trends Biochem. Sci..

[28] De Vos K., Severin F., Van Herreweghe F., Vancompernolle K., Goossens V., Hyman A., Grooten J. (2000). Tumor necrosis factor induces hyperphosphorylation of kinesin light chain and inhibits kinesin-mediated transport of mitochondria. J. Cell Biol..

[29] Yoshimura N. (1997). Cytochemical components of mitochondrial dense bodies in the brain in Menkes disease: Electron microscopic cytochemistry and X-ray microanalysis. Neuropathology.

[30] Lu J.Q., Monaco C.M.F., Hawke T.J., Yan C., Tarnopolsky M.A. (2020). Increased intra-mitochondrial lipofuscin aggregates with spherical dense body formation in mitochondrial myopathy. J. Neurol. Sci..

[31] Johnson W.T., Newman S.M. (2007). Hearts in adult offspring of copper-deficient dams exhibit decreased cytochrome c oxidase activity, increased mitochondrial hydrogen peroxide generation and enhanced formation of intracellular residual bodies. J. Nutr. Biochem..

[32] Takemura G., Kanamori H., Okada H., Tsujimoto A., Miyazaki N., Takada C., Hotta Y., Takatsu Y., Fujiwara T., Fujiwara H. (2017). Ultrastructural aspects of vacuolar degeneration of cardiomyocytes in human endomyocardial biopsies. Cardiovasc. Pathol. Off. J. Soc. Cardiovasc. Pathol..

